# High preoperative blood oxaloacetate and 2-aminoadipic acid levels are associated with postoperative delayed neurocognitive recovery

**DOI:** 10.3389/fendo.2023.1212815

**Published:** 2023-07-31

**Authors:** Haoli Mao, Huimin Huang, Ren Zhou, Jiao Zhu, Jia Yan, Hong Jiang, Lei Zhang

**Affiliations:** Department of Anesthesiology, Shanghai Ninth People’s Hospital, Shanghai Jiao Tong University School of Medicine, Shanghai, China

**Keywords:** anesthesia, delayed neurocognitive recovery, oxaloacetate, 2-aminoadipic acid, metabolomics

## Abstract

**Introduction:**

This study aimed to identify preoperative blood biomarkers related to development of delayed neurocognitive recovery (dNCR) following surgery.

**Methods:**

A total of 67 patients (≥65 years old) who underwent head and neck tumor resection under general anesthesia were assessed using the Mini-Mental State Examination (MMSE) and Montreal Cognitive Assessment (MoCA). Preoperative serum metabolomics were determined using widely targeted metabolomics technology.

**Results:**

Of the 67 patients, 25 developed dNCR and were matched to 25 randomly selected patients from the remaining 42 without dNCR. Differential metabolites were selected using the criteria of variable importance in projection > 1.0 in orthogonal partial least squares discrimination analysis, false discovery rate <0.05, and fold-change >1.2 or <0.83 to minimize false positives. Preoperative serum levels of oxaloacetate (OR: 1.054, 95% CI: 1.027–1.095, *P* = 0.001) and 2-aminoadipic acid (2-AAA) (OR: 1.181, 95% CI: 1.087–1.334, *P* = 0.001) were associated with postoperative dNCR after adjusting for anesthesia duration, education, and age. Areas under the curve for oxaloacetate and 2-AAA were 0.86 (sensitivity: 0.84, specificity: 0.88) and 0.86 (sensitivity: 0.84, specificity: 0.84), respectively. High levels of preoperative oxaloacetate and 2-AAA also were associated with postoperative decreased MoCA (β: 0.022, 95% CI: 0.005–0.04, *P* = 0.013 for oxaloacetate; β: 0.077, 95%CI: 0.016–0.137, *P* = 0.014 for 2-AAA) and MMSE (β: 0.024, 95% CI: 0.009–0.039, *P* = 0.002 for oxaloacetate; β: 0.083, 95% CI: 0.032–0.135, *P* = 0.002 for 2-AAA) scores after adjusting for age, education level, and operation time.

**Conclusion:**

High preoperative blood levels of oxaloacetate and 2-AAA were associated with increased risk of postoperative dNCR.

**Clinical trial registration:**

https://classic.clinicaltrials.gov/ct2/show/NCT05105451, identifier NCT05105451.

## 1 Introduction

Perioperative neurocognitive disorder encompasses a broad range of postoperative cognitive complications including preoperatively diagnosed cognitive decline, postoperative delirium (POD), delayed neurocognitive recovery (dNCR), and neurocognitive disorders (1, 2). dNCR refers to a decline in cognitive function approximately 1–4 weeks after anesthesia/surgery in elderly patients (1, 2). It is associated with increased risk of postoperative complications, increased length of hospital stay, or even death (3).

Identifying predictive biomarkers would help determine the individual risk of developing dNCR and could improve postoperative management of elderly patients. Although some predictive biomarkers for dNCR have been reported, such as inflammatory factors, tau protein, S100B protein, neuron-specific enolase, and brain-derived neurotrophic factor, they are mainly measured postoperatively and hence provide no preoperative predictive value (4). Assessing preoperative and postoperative risk could identify patients who are susceptible to adverse events during the perioperative period, facilitating preoperative planning and postoperative recovery. A recent study found that preoperative plasma concentrations of phosphorylated tau at threonine 217 (tau-PT217) and 181 (tau-PT181), two Alzheimer’s disease biomarkers, are associated with POD and can serve as predictive biomarkers (5). However, biomarkers that can predict dNCR are still largely unknown.

Metabolic processes in the elderly gradually change over time. Aging alters the human brain, including reduced brain weight and volume, cortical thickness, and synaptic density, as well as decreased glucose metabolism, oxygen consumption, and cerebral blood flow (6). Metabolic abnormalities in the brain are linked to neurodegenerative diseases such as mild cognitive impairment, Alzheimer’s disease, and other age-related cognitive deficits (7, 8). In the framework of systems biology based on the genome, transcriptome, proteome, and metabolome, metabolomics most closely represents biological phenotypes because it reflects biological events that occur in living organisms (9). Considering that the metabolites of all biochemical reactions in an organism contain significant information about an organism’s health, preoperative metabolites may be a useful predictive biomarker of surgical outcomes and complications in surgical patients.

Our recent study found that sevoflurane anesthesia directly alters the glucose metabolism pathway, activates glycolysis, and increases lactate levels in the brains of aged marmosets (10). Cerebrospinal fluid lactate levels are also elevated in patients with POD (11). Accumulating evidence also indicates that alteration of the perioperative metabolic pathway is closely related to POD (12, 13). These results suggest that preoperative metabolites may contribute to postoperative surgical outcomes and complications in the elderly. Therefore, we investigated whether preoperative blood metabolites in elderly individuals undergoing neck and maxillofacial tumor resection under general anesthesia were associated with development of dNCR.

## 2 Materials and methods

### 2.1 Study enrollment

This prospective observational cohort study was conducted at Shanghai Ninth People’s Hospital at Shanghai Jiao Tong University School of Medicine in Shanghai, China, in 2021–2022. After receiving approval from the Ethics Committee of Shanghai Ninth People’s Hospital (SH9H-2021-T120) and written informed consent, we recruited 67 patients aged ≥65 years who were scheduled for neck and maxillofacial tumor resection and with an American Society of Anesthesiologists physical status of I–II. Preoperative exclusion criteria included: 1) history of preoperative psychiatric disorders and psychotropic substance use; 2) diagnosis of Alzheimer’s disease; 3) abnormal preoperative assessments on the Self-Rating Anxiety Scale (score > 50), Self-Rating Depression Scale (score > 50), Mini-Mental State Examination (MMSE) [scores of <17 (illiterate), <20 (elementary education), or <24 (secondary education and above)], or Montreal Cognitive Assessment (MoCA) [score < 23 (one additional point for <12 years of education)]; or 4) participation in other clinical studies using interventional drugs. Patients were also excluded after surgery if they had: 1) history of emergency resuscitation-related illnesses in the perioperative period; 2) <2 hours of anesthesia; 3) POD [abnormal 3-minute Diagnostic Interview for CAM (3D-CAM) assessment]; or 4) early discharge, preventing completion of scale assessments and blood sampling.

This study adhered to STROBE guidelines. The study protocol was registered with clinicaltrials.gov (https://classic.clinicaltrials.gov/ct2/show/NCT05105451). Trained clinical researchers assessed participants for cognition change (primary outcome) before surgery and at 1, 3, and 7 days after surgery (between 8:00 am and 12:00 pm) using MMSE and MoCA (14). A decrease of one standard deviation (SD) in both evaluation scales after surgery was considered dNCR (15). The secondary outcome was dNCR severity, represented by decreased MoCA and MMSE scale values after surgery.

### 2.2 Anesthesia, surgery, and serum sample collection

All patients received general anesthesia under tracheal intubation. The induction drugs used were sufentanil (10–20 μg), midazolam (2 mg), propofol (1–2 mg/kg), and rocuronium (0.6 mg/kg). Preoperative antiemetics such as dolasetron and pentoxifylline hydrochloride were used as required. Intraoperative intravenous inhalation anesthesia used was sevoflurane (1.5%–2.5%) combined with propofol (2–6 mg/kg/h) and remifentanil (0.05–1 μg/kg/min). Sufentanil and rocuronium were used intermittently to deepen anesthesia and relax muscles as needed for the procedure. Patient anesthesia control was according to bleeding volume, urine volume, and central venous pressure. Timely replenishment of crystalloid (lactated or acetate Ringer’s) and colloid (hydroxyethyl starch) intravenous solutions and blood productions. We used an Avance machine (Datex-Ohmeda, Baldwin Park, CA, USA) for general anesthesia, a multifunctional monitor (DASH400, GE, Boston, MA, USA) to monitor the patient’s vital signs, and a bispectral index monitor (BeneView T5, Mindray, Shenzhen, China) to determine depth of anesthesia. Intraoperative blood gas analysis (ABL800 Flex, Radiometer, Brea, CA, USA) was used to monitor blood oxygen and adjust water–electrolyte balance. All participants received standardized perioperative care, including postoperative pain management using pentazocine (90 mg/48 h) patient-controlled analgesia.

We collected 5 mL of blood from participants before surgery and one day after surgery *via* catheterization of the dorsalis pedis artery. Collect blood and place it into the tube designed to promote coagulation (20231280, Shanghai Orsin Medical Technology Co. Ltd., Shanghai, China). Samples were incubated at 25°C for 30 min and centrifuged at 3000 rpm for 15 min at 4°C. Serum supernatant was collected and stored at -80°C.

### 2.3 Liquid chromatography–tandem mass spectrometry

According to a previous study (16), 50 µL of serum and 300 µL of methanol were mixed by vortexing for 10 min at 4°C and 2000 rpm. After centrifugation at 4°C for 10 min at 12,000 rpm, 300 µL of supernatant were transferred to a new tube. The supernatant was dried using a stream of nitrogen. The dry residue was reconstituted in 50 μL of 2% acetonitrile, mixed by vortexing at 4°C for 10 min at 2000 rpm, and centrifuged at 4°C for 10 min at 12,000 rpm. The supernatant was transferred to an autosampler vial. Sample extracts were analyzed by reversed-phase chromatography using a mobile phase of water (containing 0.1% formic acid) and acetonitrile (containing 0.1% formic acid) at a constant flow rate of 0.20 mL/min. The injection volume was 1 µL, and the temperature of the autosampler was 40°С. Chromatographic separation was achieved on an Acquity UPLC HSS T3 column (2.1 × 100 mm, 1.8 µm, Waters Corp., Milford, USA) on a Vanquish system (ThermoFisher Scientific, Waltham, MA, USA). A TSQ Altis mass spectrometer (ThermoFisher Scientific) with electrospray ionization source was operated in multiple reaction monitoring mode for mass data acquisition. The parameters of the heated electrospray ionization source were: sheath gas at 40 arbitrary units, aux gas at 10 arbitrary units, spray voltage at 3.5 kV (+)/2.5 kV (–), ion transfer tube temperature at 320°C, and vaporizer temperature at 325°C. Optimized mass spectrometer conditions were used for quantitative analysis.

### 2.4 Statistical analysis

#### 2.4.1 Clinical data

Data are expressed as mean ± SD if normally distributed; otherwise, they are expressed as median and 25^th^ and 75^th^ percentiles. Differences between groups were tested using Student’s *t*-test, Wilcoxon test, or Fisher’s exact test as appropriate. Mean and SD of patients’ preoperative MMSE and MoCA scores were calculated. dNCR was defined as a decrease of ≥1 SD on both MMSE and MoCA scales at 1, 3, or 7 days after surgery. Patients who did not develop dNCR were defined as the control group.

Receiver operating characteristic (ROC) curve analysis with R4.0.5 statistical software was used to determine differential metabolites with *P* < 0.05. Results are presented as area under the ROC curve (AUC) for metabolite cutoff, sensitivity, and specificity. AUC was calculated using the trapezoidal rule. This was used to evaluate the probability that the model would score a randomly selected positive sample higher than a randomly selected negative sample. Logistic and linear regressions were used to evaluate the association between screening indicators and occurrence of dNCR or decline in MMSE and MoCA scores. These scores were used to represent dNCR severity. Decrease in MoCA or MMSE scores was natural log-transformed for analysis. In the multivariate regression model, education level, age, and duration of anesthesia were included as covariates.

#### 2.4.2 Metabolomics analyses

Widely targeted metabolomics, based on liquid chromatography–tandem mass spectrometry technology, has diverse applications because it combines the advantages of high resolution and broad coverage of non-targeted technologies with the high sensitivity and precise quantification capabilities of targeted multiple reaction monitoring technology. Metabolomics data contained information on the abundance of 182 metabolites from 100 samples. The online analytical tool MetaboAnalyst 5.0 (https://www.metaboanalyst.ca/) was used to perform multivariate (multidimensional) statistical analysis. Multivariate statistical methods such as principal component analysis, partial least squares discrimination analysis (PLS-DA), and orthogonal PLS-DA (OPLS-DA) were used for comparative analysis of the whole metabolic spectrum and metabolite screening. Data were normalized before multivariate statistical modeling was performed. In addition to the multivariate statistical method, Student’s *t*-test and fold-change were applied to measure the significance of each metabolite. Differential metabolites were selected using criteria with OPLS-DA variable importance in projection (VIP) > 1.0, Student’s *t*-test false discovery rate (FDR) < 0.05, and fold-change of >1.2 or <0.83.

## 3 Results

### 3.1 General data and metabolomics analysis

**Figure 1:**
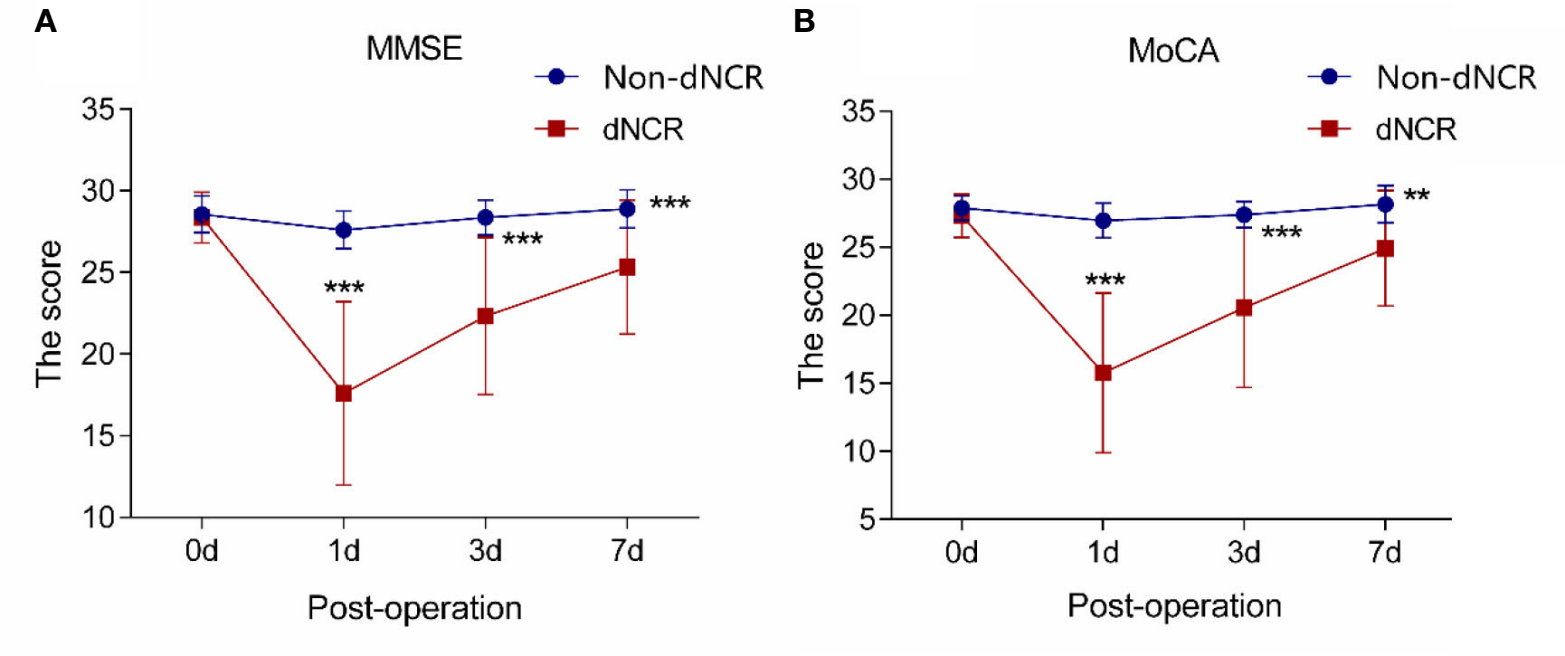
Analysis of cognitive scales in elderly patients **(A)** Analysis of Mini-Mental State Examination (MMSE) scores in 50 patients (***P < 0.001). **(B)** Analysis of Montreal Cognitive Assessment (MoCA) scores in 50 patients (**P<0.01, ***P<0.001).

Among the 67 participants recruited for this study, 25 patients developed dNCR, while 42 patients did not. Among the 42 participants without dNCR, 25 were randomly selected for matching to the 25 participants with dNCR based on sex for serum metabolomics. MMSE and MoCA test scores were significantly different between dNCR and non-dNCR groups, with the dNCR group scoring lower on both tests (**Figures 1A, B**). No significant complications occurred among participants during the postoperative period. Baseline demographic and clinical characteristics of the 50 patients are in **Table 1**. There were no significant differences in age, sex, education level, or preoperative MMSE and MoCA scores between the dNCR (n = 25) and non-dNCR (n = 25) groups. There were also no significant differences in the physiological signs and medications used during anesthesia/surgery between dNCR and non-dNCR groups (**Table 2**). Thus, metabolomics data from 50 participants were included in the final data analysis.

**Table 1:** Patients’ baseline data (N = 50).

		Non-dNCR	dNCR	*P*-value
Number of cases		25	25	
Sex (%)	Male	16 (64.0)	16 (64.0)	1
	Female	9 (36.0)	9 (36.0)	
Age (years), mean (SD)		72.00 (7.37)	73.92 (6.22)	0.3245
Education level (%)	College	4 (16.0)	2 (8.0)	0.5116
	Junior college	2 (8.0)	1 (4.0)	
	High school	4 (16.0)	2 (8.0)	
	Junior high school	6 (24.0)	10 (40.0)	
	Primary school	9 (36.0)	8 (32.0)	
	Illiterate	0 (0.0)	2 (8.0)	
Height (cm), mean (SD)		162.56 (6.46)	163.60 (7.52)	0.6024
Body weight (kg), mean (SD)		59.58 (9.11)	62.18 (9.79)	0.3359
BMI index, mean (SD)		22.46 (2.55)	23.30 (3.92)	0.3721
ASA (%)	II	25 (100.0)	25 (100.0)	
Red blood cells (×10^12^), mean (SD)		4.05 (0.64)	4.06 (0.56)	0.9684
Hemoglobin (g/L), mean (SD)		123.76 (21.26)	124.80 (15.18)	0.843
White blood cells (×10^9^), mean (SD)		33.90 (138.57)	6.88 (3.24)	0.3347
Platelet (×10^9^), mean (SD)		222.76 (55.93)	188.00 (46.01)	0.0203
Total protein (g/L), mean (SD)		68.00 (4.79)	65.68 (6.30)	0.149
Urea nitrogen (mmol/L), mean (SD)		6.47 (2.02)	7.58 (3.61)	0.1868
Creatinine (µmol/L), mean (SD)		66.32 (13.31)	69.80 (15.28)	0.3947
PT (s), mean (SD)		11.08 (0.48)	11.18 (0.81)	0.6144
APTT (s), mean (SD)		25.46 (1.63)	24.90 (2.77)	0.3878
Blood glucose (mmol/L), mean (SD)		6.18 (2.04)	5.73 (1.52)	0.3824
SAS score, mean (SD)		27.32 (2.59)	27.68 (3.45)	0.6784
SDS score, mean (SD)		28.24 (3.88)	29.52 (4.18)	0.2674
MMSE-pre score, mean (SD)		28.56 (1.12)	28.36 (1.55)	0.6037
MoCA-pre score, mean (SD)		27.88 (0.88)	27.32 (1.57)	0.1271

ASA, American Society of Anesthesiologists; PT, partial thromboplastin time; APTT, activated partial thromboplastin time; SAS, Self-Rating Anxiety Scale; SDS, Self-Rating Depression Scale; MMSE, Mini-Mental State Examination; MoCA, Montreal Cognitive Assessment.

**Table 2:** Intraoperative indicators and medication of 50 patients.

		Non-dNCR	dNCR	*P*-value
Number of cases		25	25	
SPO_2_ (%), mean (SD)		99.80 (0.58)	99.84 (0.62)	0.8151
BR (f/min), median [IQR]		12.00 [12.00, 12.00]	12.00 [12.00, 12.00]	0.2716
HR- preoperative (bpm/min), mean (SD)		71.64 (9.54)	71.24 (10.18)	0.871
HR-after induction (bpm/min), mean (SD)		69.45 (9.73)	71.20 (9.96)	0.4834
HR-intraoperative (bpm/min), mean (SD)		62.00 (11.37)	62.64 (5.52)	0.7933
HR-postoperative (bpm/min), mean (SD)		65.40 (10.32)	70.52 (11.33)	0.063
MAP-preoperative (mmHg), mean (SD)		100.23 (11.57)	104.69 (12.08)	0.1882
MAP-after induction (mmHg), mean (SD)		91.45 (11.75)	89.03 (16.89)	0.5582
MAP-intraoperative (mmHg), mean (SD)		78.36 (12.80)	77.87 (10.77)	0.8834
MAP-postoperative (mmHg), mean (SD)		87.79 (11.13)	89.27 (12.97)	0.667
BIS-preoperative, median [IQR]		98.00 [96.25, 98.00]	98.00 [97.00, 98.00]	0.7592
BIS-after induction, median [IQR]		49.00 [45.00, 50.00]	48.00 [45.00, 50.00]	0.6667
BIS-intraoperative, median [IQR]		49.00 [46.00, 52.00]	48.00 [45.00, 52.00]	0.5193
BIS-postoperative, median [IQR]		53.00 [50.00, 58.75]	53.00 [49.00, 58.00]	0.9896
Electrocardiogram (%)	N	25 (100.0)	24 (96.0)	1
	Premature pulse	0 (0.0)	1 (4.0)	
Sevoflurane inhalation concentration (%)	1.5	1 (4.0)	3 (12.0)	0.6022
	2	24 (96.0)	22 (88.0)	
Sevoflurane inhalation time, mean (SD)		5.21 (2.28)	6.03 (2.90)	0.2718
Propofol (mg), mean (SD)		901.60 (640.39)	945.88 (456.14)	0.7795
Midazolam (mg), mean (SD)		1.96 (0.20)	1.96 (0.20)	1
Rocuronium (mg), mean (SD)		56.00 (14.14)	59.80 (13.11)	0.3294
Sufentanil (µg), mean (SD)		39.60 (11.63)	39.60 (11.81)	1
Remifentanil (mg), mean (SD)		1.79 (0.74)	1.96 (0.98)	0.4921
Crystal liquid (mL), mean (SD)		2128.00 (983.41)	2128.00 (1067.13)	1
Colloidal solution (mL), mean (SD)		645.20 (413.81)	700.00 (322.75)	0.604
Blood transfusion volume (mL), mean (SD)		184.00 (310.48)	208.00 (279.76)	0.7752
Urine output (mL), mean (SD)		1272.00 (1069.86)	1208.00 (898.57)	0.8198
Bleeding volume (mL), mean (SD)		338.00 (216.16)	427.20 (236.81)	0.1706
Time of anesthesia (min), mean (SD)		350.40 (143.87)	392.32 (169.93)	0.3512
Time of operation (min), mean (SD)		322.20 (139.49)	357.92 (165.42)	0.4132

HR, heart rate; MAP, mean arterial pressure; BIS, bispectral index.

**Figure 2:**
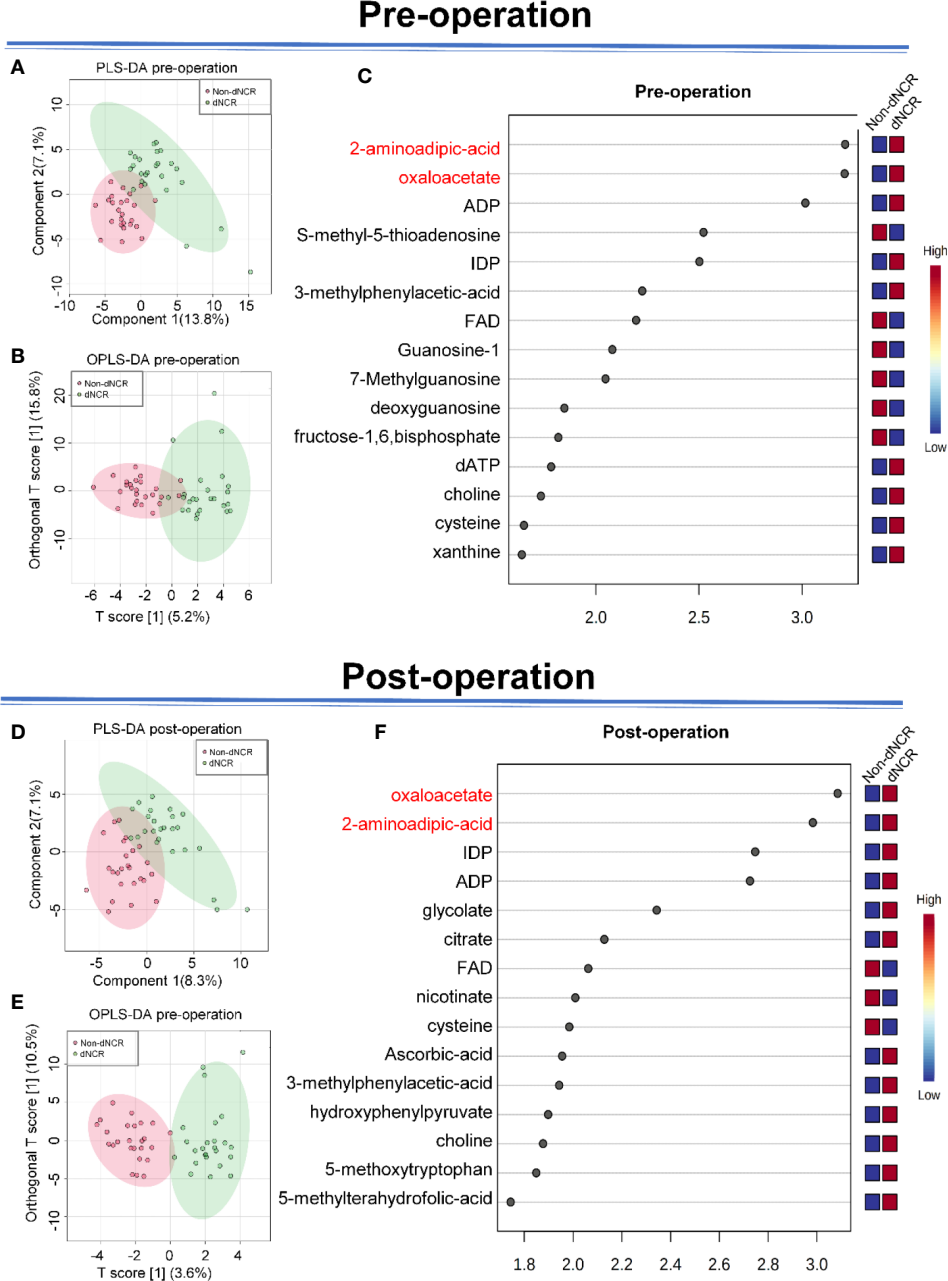
Analysis of serum metabolites in elderly patients **(A)** Partial least squares discrimination analysis (PLS-DA) of preoperative serum between delayed neurocognitive recovery (dNCR) and non-dNCR groups, component 1 (13.8%) and component 2 (7.1%). **(B)** Orthogonal PLS-DA (OPLS-DA) of preoperative serum between dNCR and non-dNCR groups, T score [1] (5.2%) and orthogonal T score [1] (15.8%). **(C)** Top 15 metabolites in variable importance in projection (VIP) map of preoperative serum (VIP > 1). **(D)** PLS-DA of postoperative serum between dNCR and non-dNCR groups, component 1 (8.3%) and component 2 (7.1%). **(E)** OPLS-DA of postoperative serum between dNCR and non-dNCR groups, T score [1] (3.6%) and orthogonal T score [1] (10.5%). **(F)** Top 15 metabolites in VIP map of postoperative serum (VIP > 1).

We first applied principal component analysis to the metabolomic data, but there was no obvious separation trend. Thus, we applied PLS-DA (**Figure 2A** and OPLS-DA (**Figure 2B**) to preoperative serum metabolomics data collected by widely targeted metabolomics (182 metabolites) between dNCR and non-dNCR groups. VIP values were used to rank the top 15 differential metabolites from OPLS-DA of preoperative serum metabolomics between dNCR and non-dNCR groups (**Figure 2C**). We also applied PLS-DA (**Figure 2D**) and OPLS-DA (**Figure 2E**) to postoperative serum metabolomics data between dNCR and non-dNCR groups. VIP values were again used to rank the top 15 differential metabolites from OPLS-DA of postoperative serum metabolomics between dNCR and non-dNCR groups (**Figure 2F**). Differential metabolites of preoperative and postoperative serum are shown in **Table 3**.

**Table 3:** Metabolites associated with dNCR in preoperative and postoperative serum (*P*_adjusted_ < 0.05, fold-change >1.2 or <0.83).

	FC	log2(FC)	*P*_adjusted_	AUC
Pre-operation				
2-AAA	1.3782	0.46274	0.000259	0.862
Oxaloacetate	1.3712	0.45548	0.000259	0.864
ADP	1.9045	0.92942	0.000281	0.870
FAD	0.65479	-0.6109	0.017134	0.792
S-methyl-5-thioadenosine	0.77791	-0.36232	0.020137	0.801
IDP	1.6603	0.73148	0.02739	0.763
3-methylphenylacetic-acid	1.2289	0.29736	0.02739	0.773
Guanosine-1	0.57193	-0.8061	0.032075	0.751
Post-operation				
Oxaloacetate	1.3768	0.46129	0.017788	0.790
2-AAA	1.3728	0.45716	0.017788	0.781
ADP	1.6243	0.69978	0.061665	0.773
FAD	0.73289	-0.44834	0.22605	0.707
S-methyl-5-thioadenosine	0.99849	-0.002183	0.99825	0.574
IDP	1.6422	0.71559	0.093066	0.744
3-methylphenylacetic-acid	1.1948	0.25673	0.22605	0.758
Guanosine-1	1.1123	0.15358	0.99091	0.518
	FC	log2(FC)	*P*.ajusted	AUC

FC, fold-change; AUC, area under curve; 2-AAA, 2-aminoadipic acid; ADP, adenosine diphosphate; FAD, flavin adenine dinucleotide; IDP, inosine diphosphate.

**Figure 3:**
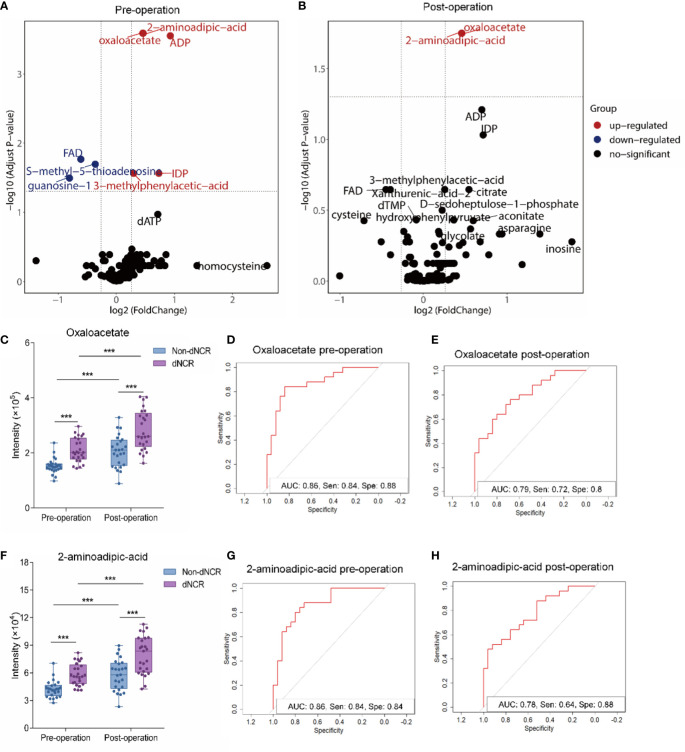
Comparative analysis of oxaloacetate and 2-aminoadipic acid (2-AAA) in serum of elderly patients before and after surgery **(A)** Volcano plot analysis of preoperative serum from delayed neurocognitive recovery (dNCR) and non-dNCR groups. Red dot represents significantly increased metabolite between dNCR and non-dNCR groups (fold-change > 1.2, *P* < 0.05). Blue dot represents significantly reduced metabolite between dNCR and non-dNCR groups (fold-change < 0.83, *P* < 0.05). **(B)** Volcano plot analysis of postoperative serum from dNCR and non-dNCR groups. Red dot represents significantly increased metabolite between dNCR and non-dNCR groups (fold-change > 1.2, *P* < 0.05). Blue dot represents significantly reduced metabolite between dNCR and non-dNCR groups (fold-change < 0.83, *P* < 0.05). **(C)** Analysis of oxaloacetate in preoperative and postoperative serum between dNCR and non-dNCR groups (***P < 0.001). **(D)** Preoperative receiver operating characteristic (ROC) curve of oxaloacetate [area under the curve (AUC): 0.86, sensitivity: 0.84, specificity: 0.88]. **(E)** Postoperative ROC curve of oxaloacetate (AUC: 0.79, sensitivity: 0.72, specificity: 0.80). **(F)** Analysis of 2-AAA in preoperative and postoperative serum between dNCR and non-dNCR groups (***P < 0.001). **(G)** Preoperative ROC curve of 2-AAA (AUC: 0.86, sensitivity: 0.84, specificity: 0.84). **(H)** Postoperative ROC curve of 2-AAA (AUC: 0.78, sensitivity: 0.64, specificity: 0.88).

FDR assesses the confidence of large-scale metabolomics data. The criteria of fold-change >1.2 or <0.83 and *P*_adjusted_ < 0.05 were used to make multiple comparison adjustments in preoperative and postoperative serum metabolomics data between dNCR and non-dNCR groups. In preoperative serum metabolomics, we found eight differential metabolites between dNCR and non-dNCR groups. Among of them, oxaloacetate and 2-aminoadipic acid (2-AAA) exhibited the most remarkable differences (**Table 3**, **Figure 3A**). In postoperative serum metabolomics, the only differential metabolites identified between dNCR and non-dNCR groups were oxaloacetate and 2-AAA (**Table 3**, **Figure 3B**).

### 3.2 Preoperative and postoperative serum oxaloacetate and 2-AAA levels associated with postoperative dNCR

Serum oxaloacetate levels significantly differed between dNCR and non-dNCR groups before and after surgery (**Figure 3C**) (*P* < 0.001), and both groups had significantly higher levels post-surgery compared to pre-surgery (*P* < 0.001). Oxaloacetate had a preoperative AUC of 0.86 (sensitivity: 0.84, specificity: 0.88) and postoperative AUC of 0.79 (sensitivity: 0.72, specificity: 0.80) (**Figures 3D, E**). Serum 2-AAA levels also significantly differed between dNCR and non-dNCR groups before and after surgery (**Figure 3F**) (*P* < 0.001), and both groups had significantly higher levels post-surgery compared to pre-surgery (*P* < 0.001). 2-AAA had a preoperative AUC of 0.86 (sensitivity: 0.84, specificity: 0.84) and postoperative AUC of 0.78 (sensitivity: 0.64, specificity: 0.88) (**Figures 3G, H**).

### 3.3 Preoperative and postoperative serum oxaloacetate and 2-AAA levels associated with severity of postoperative dNCR

Data with adjusted and unadjusted analyses and multivariate models are in **Table 4**. After adjustment for anesthesia duration, education, and age, preoperative serum levels of oxaloacetate and 2-AAA were significantly associated with postoperative dNCR in separate models.

**Table 4:** Oxaloacetate and 2-aminoadipic acid (2-AAA) in preoperative serum significantly predict dNCR presence and severity.

dNCR presence*				
	Unadjusted		Adjusted for operation time, education, and age	
	OR (95% CI)	*P*	OR (95% CI)	*P*
Oxaloacetate (per 1000 units)	1.048 (1.024–1.082)	< 0.001	1.054 (1.027–1.095)	0.001
2-AAA (per 1000 units)	1.1619 (1.079–1.286)	< 0.001	1.181 (1.087–1.334)	0.001
dNCR severity, measured by decreasing MMSE score**				
	Unadjusted		Adjusted for operation time, education, and age	
	β (95% CI)	*P*	β (95% CI)	*P*
Oxaloacetate (per 1000 units)	0.024 (0.01–0.038)	0.001	0.024 (0.009–0.039)	0.002
2-AAA (per 1000 units)	0.084 (0.034–0.133)	0.001	0.083 (0.032–0.135)	0.002
dNCR severity, measured by decreasing MoCA score***				
	Unadjusted		Adjusted for operation time, education, and age	
	β (95% CI)	*P*	β (95% CI)	*P*
Oxaloacetate (per 1000 units)	0.024 (0.007–0.041)	0.006	0.022 (0.005–0.04)	0.013
2-AAA (per 1000 units)	0.082 (0.022–0.1420	0.008	0.077 (0.016–0.137)	0.014

*Models adjusted for associations between biomarkers and outcomes by operation time, education, and age, based on clinically relevant previous studies. Results are presented as odds ratio (OR) per 1000-unit change in oxaloacetate or 2-AAA value and associated 95% confidence interval (CI) with null hypothesis of 1.

**Results presented as beta coefficient (β) per 1000-unit change in oxaloacetate or 2-AAA value and associated 95% CI with null hypothesis of 0, related to MMSE score.

***Results presented as beta coefficient (β) per 1000-unit change in oxaloacetate or 2-AAA value and associated 95% CI with null hypothesis of 0, related to MoCA score.

In unadjusted models, preoperative higher serum oxaloacetate and 2-AAA levels were associated with increased dNCR severity, as measured by degree of decrease in MMSE and MoCA scores. This association persisted after adjustment for preoperative MMSE score. There also was a strong association after adjustment for preoperative MoCA (**Table 4**).

After adjustment for operation time, education, and age, postoperative serum levels of oxaloacetate and 2-AAA also were associated with postoperative dNCR in separate models. This association also persisted after adjustment for postoperative MMSE score. There also was a strong association after adjustment for postoperative MoCA score (**Table 5**).

**Table 5:** Oxaloacetate and 2-aminoadipic acid (2-AAA) in postoperative serum significantly predict dNCR presence and severity.

dNCR presence*				
	Unadjusted		Adjusted for operation time, education, and age	
	OR (95% CI)	*P*	OR (95% CI)	*P*
Oxaloacetate (per 1000 units)	1.019 (1.008–1.034)	0.002	1.019 (1.008–1.034)	0.002
2-AAA (per 1000 units)	1.063 (1.026–1.112)	0.002	1.063 (1.026–1.112)	0.002
dNCR severity, measured by decreasing MMSE score**				
	Unadjusted		Adjusted for operation time, education, and age	
	β (95% CI)	*P*	β (95% CI)	*P*
Oxaloacetate (per 1000 units)	0.044 (0.021–0.067)	0.001	0.044 (0.021–0.067)	< 0.001
2-AAA (per 1000 units)	0.152 (0.07–0.234)	0.002	0.152 (0.07–0.234)	< 0.001
dNCR severity, measured by decreasing MoCA score***				
	Unadjusted		Adjusted for operation time, education, and age	
	β (95% CI)	*P*	β (95% CI)	*P*
Oxaloacetate (per 1000 units)	0.04 (0.014–0.065)	0.007	0.04 (0.014–0.065)	0.004
2-AAA (per 1000 units)	0.138 (0.047–0.23)	0.009	0.138 (0.047–0.23)	0.005

*Models adjusted for associations between biomarkers and outcomes by operation time, education, and age, based on clinically relevant previous studies. Results are presented as odds ratio (OR) per 1000-unit change in oxaloacetate or 2-AAA value and associated 95% confidence interval (CI) with null hypothesis of 1.

**Results presented as beta coefficient (β) per 1000-unit change in oxaloacetate or 2-AAA value and associated 95% CI with null hypothesis of 0, related to MMSE score.

***Results presented as beta coefficient (β) per 1000-unit change in oxaloacetate or 2-AAA value and associated 95% CI with null hypothesis of 0, related to MoCA score.

## 4 Discussion

This study demonstrated that patients with higher preoperative levels of oxaloacetate or 2-AAA are more likely to experience dNCR and have more severe dNCR following surgery. These data suggest that preoperative metabolites of oxaloacetate or 2-AAA may be used to identify patients at risk of dNCR.

It is interesting to note that oxaloacetate levels of both dNCR and non-dNCR groups were increased after anesthesia/surgery. We speculate that anesthesia/surgery may increase preoperative oxaloacetate and 2-AAA levels to reach a certain threshold that determines dNCR. In addition, patients who develop POD also have elevated oxaloacetate levels (13). Together, these results suggest oxaloacetate may serve as a biomarker that can predict perioperative neurocognitive disorder. This may occur *via* an imbalance in energy homeostasis, as oxaloacetate is a metabolism substrate in the tricarboxylic acid cycle. Consistently, glycolysis but not oxidative phosphorylation is enhanced in the brain of aged mice and marmosets after anesthesia/surgery (10, 17).

We measured increased levels of key metabolites both before and after surgery. Preoperative biomarkers have clear predicative power. Yet despite lacking predictive preoperative power, postoperative measures are still clinically valuable. First, it is important to establish baselines values for metabolite levels both pre- and post-surgery to inform how these levels change in response to surgery/anesthesia. In addition, clinical interventions both pre- and post-surgery can help ameliorate dNCR—preoperative interventions can help predict occurrence and reduce the frequency of dNCR, while postoperative interventions can help reduce the degree of dNCR if it does occur.

Surgical trauma induces systemic inflammatory responses and subsequent neuroinflammation (3). A clinical study reported that cardiac surgery triggers systemic inflammation and neuroinflammation, reduces key brain regional functional connectivity, and impairs neurological function (18). In addition, gut injury following orthopedic surgery causes gut microbiome dysbiosis and intestinal barrier dysfunction, which synergistically results in severe systematic neuroinflammation that damages brain function, especially in prodromal Alzheimer’s disease patients (19). Neuroinflammation can cause energy deficiency and modifications in metabolic pathways in the brain, contributing to brain aging and cognitive decline connected to Alzheimer’s disease (20). Although we do not know if such changes occurred in our patients, those changes can cause metabolic imbalance.

We found both oxaloacetate and 2-AAA were associated with presence and severity of dNCR. Oxaloacetate is a key metabolism substrate in the tricarboxylic acid cycle, and 2-AAA is generated by lysine degradation and may serve as a substrate for downstream enzymes of tryptophan metabolism. Previous studies demonstrate that POD is associated with inhibition of the citric acid cycle (13) and activation of the tryptophan pathway (21). Our findings suggest that POD and DNCR may share a common mechanism.

Further, 2-AAA is directly toxic to astrocytes (22–25). Animal experiments show that injection of 2-AAA into the medial prefrontal cortex causes loss of astrocytes, triggering neuronal damage and resulting in cognitive impairment (26). Interestingly, 2-AAA leads to astrocyte degeneration in the striatum, without affecting neurons or striatal fibers, which ameliorates dopaminergic neurodegeneration and dyskinesia in a male rat model of Parkinson’s disease inflammation (27, 28). Moreover, 2-AAA is a predictive biomarker for diabetes risk and insulin resistance (29, 30) and also serves as a reliable biomarker for protein oxidation (31, 32). In central nervous system diseases, increased 2-AAA levels are closely related to impaired cognitive function in the elderly, especially those with mild cognitive impairment and Alzheimer’s disease (33, 34).

Despite its strengths, our study has a few limitations. First, the predictive power of the two identified metabolites is not dramatic. However, this study revealed a high frequency (37%) of dNCR in patients undergoing head, neck, and maxillofacial surgery. Previous studies have indicated that dNCR incidence is ~40% in cardiac surgery patients (35) and 15% (36)–34% (37) in non-cardiac surgery patients. Moreover, oxaloacetate is already reported to be associated with other perioperative complications such as POD (13), highlighting the clinical value of our study. Second, considering our sample size was small and came from a single large hospital, it would be beneficial to replicate our findings with larger, more diverse samples from patients with other cancer types and/or non-cancer patients undergoing surgery.

In conclusion, our study found that patients with dNCR had higher preoperative serum oxaloacetate or 2-AAA levels. These biomarkers were closely associated with dNCR incidence and severity. Our data further suggest that preoperative metabolism disorder is already present in patients with high risk of developing neurological complications following surgery. Further research is necessary to ascertain the presence of oxaloacetate or 2-AAA as biomarkers in other types of cancer as well as in non-cancer patients who are undergoing surgery. Considering that oxaloacetate is a key metabolite of the citric acid cycle and tryptophan metabolism is related to 2-AAA metabolism, dNCR intervention may focus on adjusting energy supply and tryptophan metabolism during the perioperative period. Our results thus prompt additional research to further understand the mechanisms underlying the interaction of metabolism and dNCR.

## Data availability statement

The original contributions presented in the study are included in the article/supplementary materials. Further inquiries can be directed to the corresponding authors.

## Ethics statement

The studies involving human participants were reviewed and approved by The Ethics Committee of Shanghai Ninth People’s Hospital. The patients/participants provided their written informed consent to participate in this study.

## Author contributions

LZ: conceptualization, investigation, writing – reviewing and editing, funding acquisition. HJ: conceptualization, methodology, funding acquisition. HM: data curation, investigation, formal analysis, writing – original draft preparation. HH: data curation, investigation, formal analysis. RZ: software, validation. JZ: software, validation. JY: data curation, investigation. All authors contributed to the article and approved the submitted version.
